# Strongyloidiasis gastritis and colitis in a patient with leprosy

**DOI:** 10.1590/0037-8682-0189-2022

**Published:** 2022-09-19

**Authors:** Francisco Kennedy Scofoni Faleiros de Azevedo, Paula Maria Pinheiro Miranda, Ivana Menezes

**Affiliations:** 1 Universidade Federal de Mato Grosso, Faculdade de Medicina, Hospital Universitário Júlio Muller, Departamento de Clínica Médica, Cuiabá, MT, Brasil.; 2 Universidade Federal de Mato Grosso, Faculdade de Medicina, Hospital Universitário Júlio Muller, Serviço de Patologia, Cuiabá, MT, Brasil.

**FIGURE 1:**
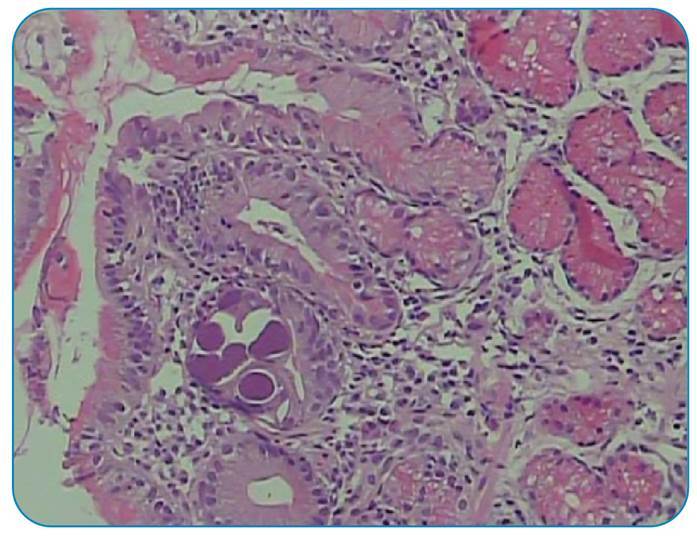
Pathological examination of a gastric tissue fragment showing the mucosa with a histologically normal fundus, parasitic structures compatible with *Strongyloides stercoralis*, and absence of neoplasia or *Helicobacter pylori.*

**FIGURE 2:**
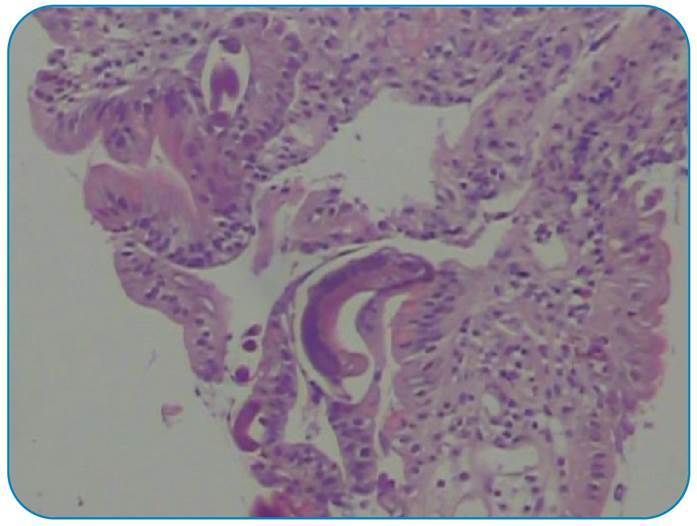
Pathological examination of the duodenum fragment showing colonic pattern mucosa with discrete colitis, parasitic structures compatible with *Strongyloides stercoralis*, and absence of neoplasm.

**FIGURE 3:**
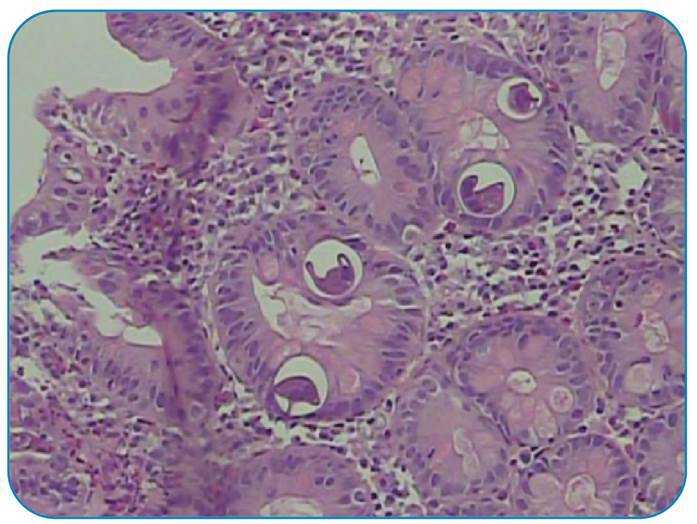
Pathological examination of ascending colon fragment showing colonic pattern mucosa with discrete colitis, presence of eosinophils and parasitic structures compatible with *Strongyloides stercoralis*, and absence of neoplasm.

A 45-year-old male patient from the Central West region of Brazil was treated for lepromatous (or virchowian hanseniasis) multibacillary leprosy 7 years ago with dapsone, clofazimine, and rifampicin for 2 years. Intermittent administration of thalidomide (300 mg/day) and prednisone (60 mg/day) for 2 years was required for the episodes of erythema nodosum. Eighteen months ago, leprosy treatment was restarted with ofloxacin, clofazimine, and rifampicin, along with thalidomide and prednisone. The patient was hospitalized with a chief complaint of abdominal pain for 2 months and weight loss of 15 kg. Upper gastrointestinal endoscopy and colonoscopy performed during the clinical investigation revealed moderate gastritis, duodenitis, and mild pancolitis. Anatomopathological analysis showed the presence of *Strongyloides stercoralis* in the gastric (Figure 1), duodenal (Figure 2), and colonic (Figure 3) fragments. The patient’s condition improved clinically after initial treatment with ivermectin and later with thiabendazole. The patient was discharged with outpatient follow-up.

Disseminated strongyloidiasis in patients with intermittent leprosy reactions and chronic steroid use should be considered^1^ ^,^ ^2^.
